# Exogenous Galectin-9 prevents peri-implantitis in rats by regulating macrophage polarization

**DOI:** 10.3389/fphar.2025.1533468

**Published:** 2025-02-28

**Authors:** Lujin Cheng, Xiaowei Ji, Huerxidai Yilihamujiang, Shuya Dong, Long Mei, Guo Lin, Qihan Tang, Zhongcheng Gong

**Affiliations:** ^1^ Department of Prosthodontics and Dental Implant, The First Affiliated Hospital of Xinjiang Medical University (Affiliated Stomatology Hospital), Urumqi, China; ^2^ Stomatological Research Institute of Xinjiang Uygur Autonomous Region, Urumqi, China; ^3^ School of Stomatology, Xinjiang Medical University, Urumqi, China; ^4^ Oncological Department of Oral and Maxillofacial Surgery, The First Affiliated Hospital of Xinjiang Medical University, School/Hospital of Stomatology Xinjiang Medical University, Urumqi, China

**Keywords:** peri-implantitis, rat, Galectin-9, macrophage polarization, alveolar bone resorption

## Abstract

**Background:**

Peri-implantitis (PI) is a common complication of oral implants with no definitive treatment strategy. Lipopolysaccharides (LPS) are involved in PI by activating macrophages and influencing osteoclasts and osteoblasts. Galactin-9 (Gal-9) is known for its immunomodulatory properties and interactions with macrophage polarization receptors. This study investigated the mechanism of prophylactic exogenous Gal-9 in the prevention and treatment of PI in rats.

**Methods:**

Male SD rats with titanium implants were divided into the Control, Saline, and Gal-9 groups. Rats in the Saline group and Gal-9 group were prophylactically administered Gal-9 and Saline, respectively, before inducing PI. Periodontal examinations, X-ray imaging, flow cytometry analyses, and micro-CT evaluations were conducted to assess clinical, imaging, and immunological parameters.

**Results:**

After inducing PI, the implant loss rate in the Gal-9 group was lower than that in the Saline group. The gingival index was higher in the Saline group and Gal-9 group compared to the Control group. The bleeding on probing positivity rate was higher in the Saline group than in the other two groups. X-ray and micro-CT images both showed lower alveolar bone resorption in the Gal-9 group than in the Saline group. Flow cytometry showed that the proportions of M1-type macrophages and M2-type macrophages, and M1/M2 ratio were lower in the Gal-9 group than those in the Saline group. Multivariate linear regression indicated that Tb.Th had the greatest impact on the gingival index and Tb.Sp had the greatest impact on the M1/M2 ratio.

**Conclusion:**

Exogenous Gal-9 administration demonstrated promising effects in mitigating inflammation associated with PI in rat models by promoting M2 macrophage polarization and enhancing alveolar bone stability.

## 1 Introduction

Peri-implant diseases are one of the main complications associated with oral implants, including peri-implant mucositis peri-implant mucositis, and peri-implantitis (PI) (Lee et al., 2017). Peri-implant mucositis is a reversible inflammation that does not cause bone resorption and can be cured and reversed through pharmacological prevention or treatment. In contrast, PI refers to the inflammatory lesions of peri-implant mucosa accompanied by irreversible loss of supporting bone, often resulting from the progression of peri-implant mucositis. Even with pharmacological treatment, PI frequently recurs, impacting the long-term success rate of implants (Heitz-Mayfield, 2008). Therefore, while seeking effective methods to treat PI, we should focus more on how to prevent its occurrence or treat peri-implant mucositis to avoid further irreversible bone resorption, which presents a challenging issue in clinical practice.

Currently, there is a lack of consensus regarding the optimal strategies for preventing and treating PI. In clinical practice, lipopolysaccharides (LPS) derived from Gram-negative bacteria are widely recognized as crucial initiators of PI (de Waal et al., 2017; Yilihamujiang et al., 2024). LPS can enter the peripheral blood through gingival crevicular fluid and then activate macrophages (Li et al., 2023). Macrophages play a pivotal role in regulating the functions of osteoclasts and osteoblasts by synthesizing and releasing a diverse array of cytokines and inflammatory mediators. Specifically, the M1 subset promotes inflammatory responses and pathogen clearance, whereas the M2 subset limits excessive inflammation and facilitates tissue repair (Ge et al., 2024). The two phenotypes of macrophages are in dynamic balance and are regulated by different cytokines and upstream/downstream signaling pathways, thereby affecting the progression of PI (Li et al., 2023).

Galectin-9 (Gal-9) is a member of the β-galactoside-binding lectin family, recognized for its potential immunomodulatory and therapeutic applications, and is present in both humans and rats (Gossink et al., 2025). It plays a role in regulating immune responses, suppressing inflammation, and maintaining immune balance. T cell immunoglobulin and mucin-domain-containing molecule-3 (Tim-3) is a key member of the costimulatory family and plays an important role in maintaining autoimmune tolerance. This is achieved through its interaction with the ligand Gal-9, which modulates the activity of inflammatory cells and maintains immune balance (Cheng and Ruan, 2015). Tim-3 is an important molecule in regulating macrophage polarization (Yoshida et al., 2024). Currently, Gal-9 has shown good therapeutic effects in diseases such as lymphoma, autoimmune arthritis, experimental autoimmune encephalomyelitis, and some infectious diseases (Seki et al., 2008; Kanzaki et al., 2012; Oomizu et al., 2012; Dobano-Lopez et al., 2024; Teng et al., 2024). It has been reported that Gal-9 activates the Tim-3 on bone marrow-derived macrophages to promote M2 polarization (Das et al., 2015). Blocking Gal-9/Tim-3 can significantly inhibit M2 polarization and angiogenesis (Ni et al., 2022), contributing to inflammation reduction and stability of the alveolar bone. In addition, regulating macrophages towards M2 polarization not only alleviates inflammation but also plays a crucial role in maintaining the stability of alveolar bone (Li et al., 2023). However, whether the regulation of macrophage polarization by Gal-9 protein can affect the outcome of PI remains to be further determined.

Herein, this research aims to elucidate the mechanism by which exogenous Gal-9 influences the prevention and treatment of PI in rat models. Preventive approaches are crucial in managing peri-implant diseases, given the high incidence of morbidity associated with PI once it has progressed (Wu et al., 2021). While Gal-9 may reduce inflammation during established PI (Gossink et al., 2025), treatment during disease progression cannot reverse the irreversible bone loss that often accompanies this condition. Therefore, we explored the potential of prophylactic Gal-9 administration to maintain bone integrity and delay or prevent disease onset. Comprehensive analyses of clinical, imaging, and immunological indicators were conducted to evaluate macrophage polarization, inflammatory responses, and alveolar bone stability. Our findings shed light on the potential of exogenous Gal-9 protein as a therapeutic agent for mitigating the inflammation associated with PI in rats, thereby offering a promising approach for the prevention and treatment of PI.

## 2 Materials and methods

### 2.1 Study animals

Male SD rats (n = 84; specific pathogen-free grade; weighing between 450 and 500 g; 5–6 months old) were purchased from the Animal Experiment Center of Xinjiang Medical University. These rats were housed in the specific pathogen-free animal facility and maintained under controlled conditions at a room temperature of 20°C–22°C. They were exposed to a 12-h light-dark cycle (lights on at 8:00 and off at 20:00) and had free access to food and water. The invasive procedures were performed under anesthesia by administering an intramuscular injection of a solution consisting of Zoletil 50 (Virbac, Milano, Italy) and Xylazine (Dunhuang Shengda Animal Medicine Co., Ltd., Dunhuang, China) at a 2:1 ratio, with a dosage of 0.1 mL/kg. All animal experiments received approval from the Ethics Committee of Approval Letter of Animal Ethics Committee of First Affiliated Hospital of Xinjiang Medical University (approval no. 20220309-193).

### 2.2 Establishment of the implant model

After acclimation for 1 week, a titanium implant (Xi’an Kangtuo Medical Technology Co., Ltd., Xi’an, China) measuring 1.6 mm in diameter and 4 mm in length was surgically placed at the mesial position of the first molar on the right maxilla. The implant surface was prepared using sandblasting and acid etching techniques. Post-implantation, X-ray images were obtained, and a subcutaneous injection of 0.2 mL/kg cefuroxime sodium (Shandong Runze Pharmaceutical Co., Ltd.) was administered to prevent infection. Weekly monitoring of rat weight and health status, including observation for hair loss, fever, and tremors, was conducted.

### 2.3 Animal grouping and treatment

At the 6th week after implantation (Blank et al., 2021), X-ray images of rats with titanium implants were taken. The rats were further screened with specific criteria. Inclusion criteria: 1) The rats survived until the end of the experiment without any signs of illness, such as weight loss, increased temperature, fur changes, or trembling. 2) The implants were placed at the mesial position of the first molar on the right maxilla (Yue et al., 2020). Exclusion criteria: 1) Incorrect implantation sites, such as the maxillary palatal area or buccal side of the first molar. 2) Absence of bone integration. 3) Implant loss. 4) Rats of natural death. According to these criteria, 18 rats were selected and randomly divided into 3 groups: Control group (n = 6), Saline group (n = 6), and Gal-9 group (n = 6). A 4-week drug intervention was conducted as follows: the Saline group received normal saline dripped around the implant gap at 5 μL per administration, twice daily (morning and evening); the Gal-9 group received recombinant human Gal-9 protein (1 mg/mL) (AmyJet Scientific Inc., Wuhan, China) at 5 μL per administration, twice daily (morning and evening); and the Control group did not undergo any intervention.

### 2.4 Establishment of the PI model

After soaking the silk sutures (4–0) in the suspension of LPS (Sigma-Aldrich, 1 mg/mL) for 30 min, they were tied around the neck of implants in the Saline group and Gal-9 group. Daily injections of LPS were administered around the implant gaps, once a day, 10 μL/day. During this period, periodontal examinations were conducted weekly, and X-ray images were taken after 6 weeks to confirm the successful establishment of the PI model.

### 2.5 Periodontal examination

The bleeding on probing (BOP) index was recorded using a force of 0.245 N to probe four sites (the cheek, tongue, mesial, and distal sides) of the implant following expert consensus (Mombelli et al., 1995). The Gingival Index (GI) score was recorded based on the following criteria: 0 points for healthy mucosa around the implant, 1 point for mild mucosal inflammation with slight color change and no bleeding upon probing, 2 points for moderate mucosal inflammation, with redness, swelling, and bleeding on probing, and 3 points for severe mucosal inflammation with obvious redness, swelling, and a tendency for spontaneous bleeding (Mombelli et al., 1995).

### 2.6 X-ray examination

X-ray images were taken at weeks 0, 6, 10, and 16 to measure the length of bone absorption. All images were captured by the same researcher on rats in the same direction, angle, and position to reduce errors.

### 2.7 Sample collection

At the end of week 16, peripheral blood samples (1–2 mL) were collected from the tail vein and anticoagulated using EDTA (1.8 mg/mL). Then, rats were euthanized by cervical dislocation under glutaraldehyde anesthesia. The maxillae of the rats were dissected and then fixed in 10% formalin.

### 2.8 Flow cytometry

The anticoagulant peripheral blood was added to the sample tube (100 uL each), in which the white blood cell concentration reached 2 × 10^6^ cells/mL after red blood cell lysis. Subsequently, the following monoclonal antibodies were added: PE/Cy7 Mouse Anti-Rat CD45 (#202213; Biolegend, San Diego, CA, United States), PE Mouse Anti-Rat F4/80 (#SC-377099; Santa, Santa Cruz, CA, United States), FITC Mouse Anti-Rat CD163 (#Bs-2527R-FITC; Bioss, Wuhan, China), and APC Mouse Anti-Rat CD86 (#200315; Biolegend), followed by incubation in the dark at room temperature for 15–30 min. These antibodies were ready-to-use formulations, with 5 µL of each antibody added to each sample. Then, 2 mL of 1× red blood cell lysis solution was added to every 100 uL of the blood samples, followed by incubation at room temperature for 8–12 min until the cell suspension turned clear and transparent. After red blood cell lysis, centrifugation was performed at 500 × g for 5 min, followed by cell washing and removal of the supernatant. Subsequently, the samples were suspended in 500 uL of PBS and analyzed by flow cytometry. The CD45^+^F4/80^+^CD86^+^ cells were gated as the M1 macrophages, while the CD45^+^F4/80^+^CD163^+^ cells were defined as M2 macrophages.

### 2.9 Micro-CT

The maxilla bone of rats was fixed for imaging purposes. The micro-CT images were taken perpendicular to the long axis of the implant using the AX2000 system (Always Imaging Industrial CT Technology, Shanghai, China). Subsequently, the obtained images were analyzed using ImageJ software. All images were calibrated based on the known lengths of the implants, and measurements were taken for trabecular number (TB.N), trabecular separation (Tb.Sp), bone volume/total volume (BV/TV), and trabecular thickness (Tb.Th).

### 2.10 Statistical analysis

Data were processed using SPSS version 22.0 (SPSS Inc., Chicago, IL, USA). Continuous variables are expressed as mean ± standard deviation. The one-way analysis of variance was conducted to compare the results among three groups, followed by the Bonferroni correction for *post hoc* analysis. Repeated measures analysis of variance was conducted to examine the same indicator at different time points, followed by Bonferroni correction. Count data are presented as percentages, and group comparisons were performed using the chi-square test. Bivariate Spearman’s rank correlation was applied to determine simple correlations, with the dependent variables of GI value and M1/M2 ratio. For the influence of multiple independent variables on dependent variables, multivariate linear regression was used to analyze the correlation between independent variables and dependent variables, as well as the potential interaction between independent variables. Graphs were plotted using GraphPad Prism version 9.4. All statistical analyses were conducted at a significance level of α = 0.05.

## 3 Results

### 3.1 Induction of the PI model

**FIGURE 1:**
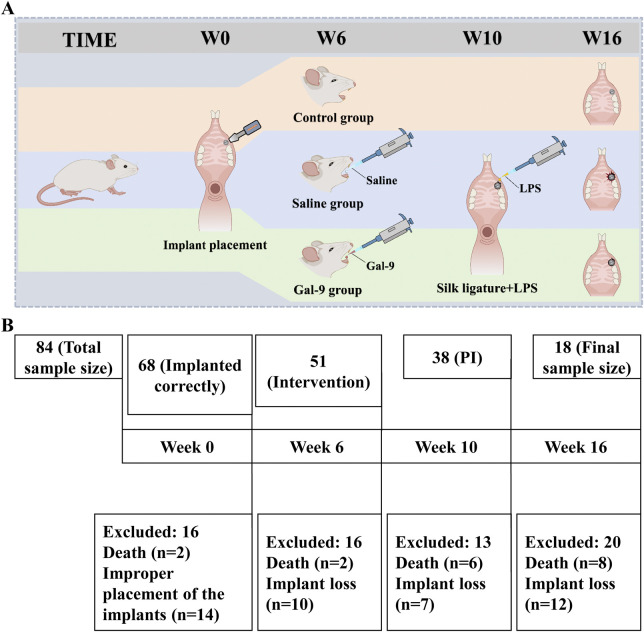
Schematic diagram of implantation and PI induction from 0 to 16 weeks. **(A)** Induction process of PI in rats. **(B)** Detailed fishbone chart of sample loss during PI induction.

**FIGURE 2:**
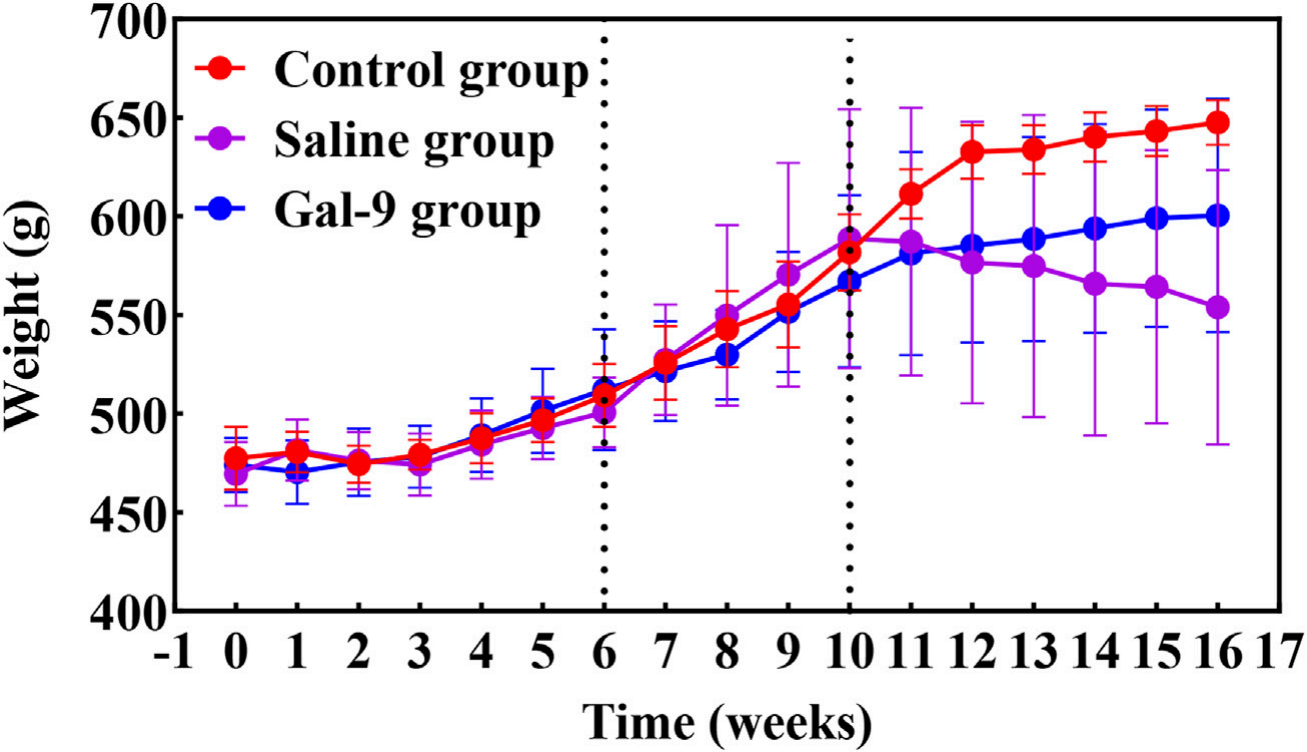
Analysis of body weight change of SD rats in each group.

The study flowchart for implant placement, drug intervention, and PI model establishment is presented in Figure 1A. A total of 84 implants were implanted into the oral cavity of rats. Ultimately, only 18 rats that met the inclusion criteria were included in this study and subjected to drug intervention and PI model establishment. A schematic of animal inclusion and exclusion is shown in Figure 1B. The implant loss rate of the Saline group increased from 5.3% to 31.3% after induction of PI (*P* < 0.05) (Table 1). The changes in rat body weight from 0 to 16 weeks are illustrated in Figure 2. The body weight of the implants increased steadily from weeks 0–10. From the 10th week onwards, both the body weight of the Control group and the Gal-9 group showed a continuous upward trend, while the Saline group exhibited a downward trend in weight. However, no significant differences were observed among the three groups (*P* > 0.05).

**TABLE 1:** Analysis of implant loss rate.

Implant loss	Control group	Saline group	Gal-9 group	χ^2^	*P*-value
Before PI	4/17 (23.5%)	1/17 (5.9%)	2/17 (11.8%)	2.318	0.314
After PI	3/12 (25%)	6/15 (31.3%)	3/11 (27.3%)	0.827	0.661
χ^2^	0.008	5.428	1.095		
*P*	0.927	0.02	0.295		

Note: PI, peri-implantitis; Gal-9, Galactin-9.

### 3.2 Comparative analysis of GI and BOP in PI-induced rats

**FIGURE 3:**
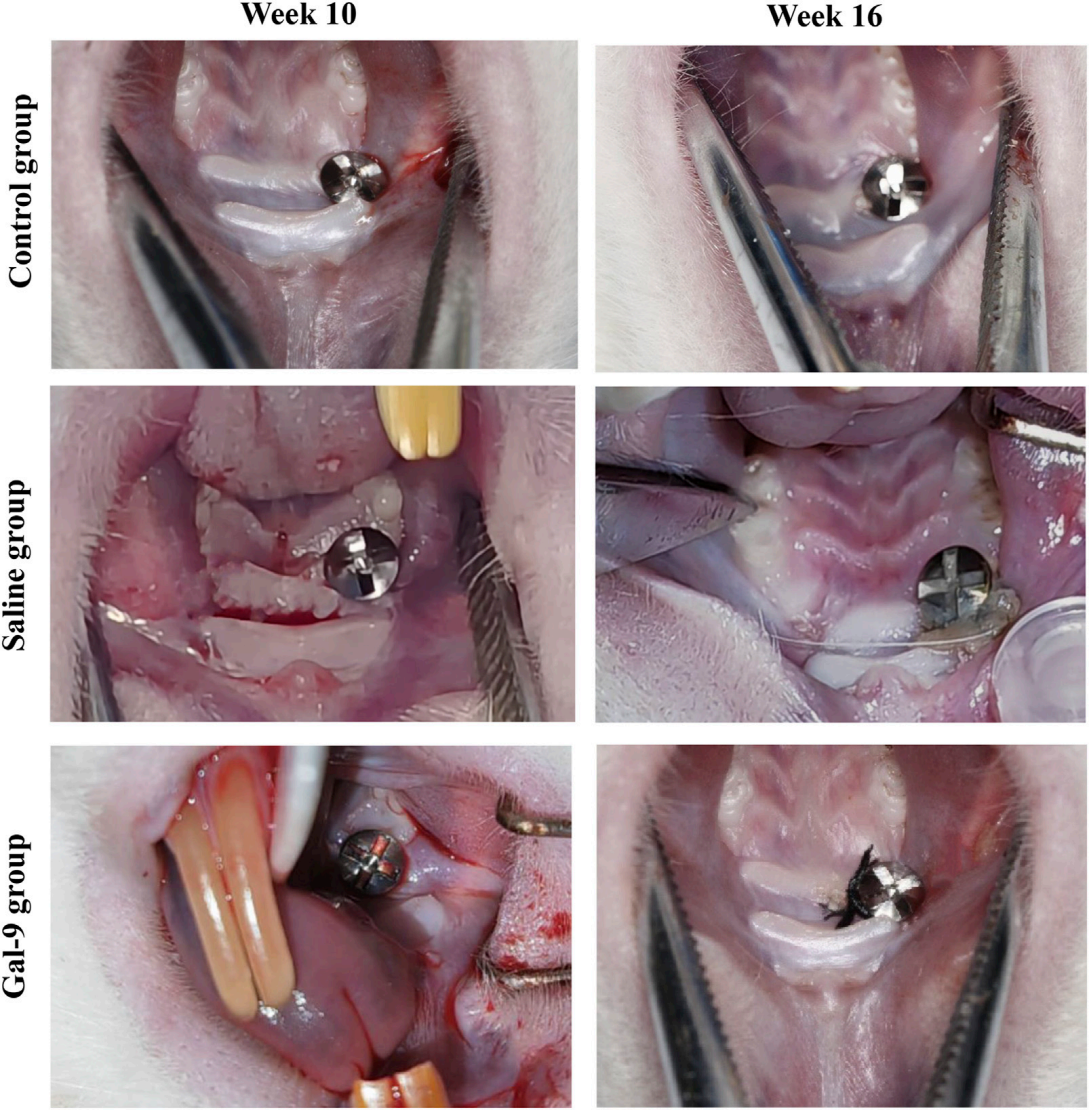
Dental images before and after PI induction. The images at week 10 (before PI) and week 16 (after PI) in the Saline and Gal-9 groups are presented.

**FIGURE 4:**
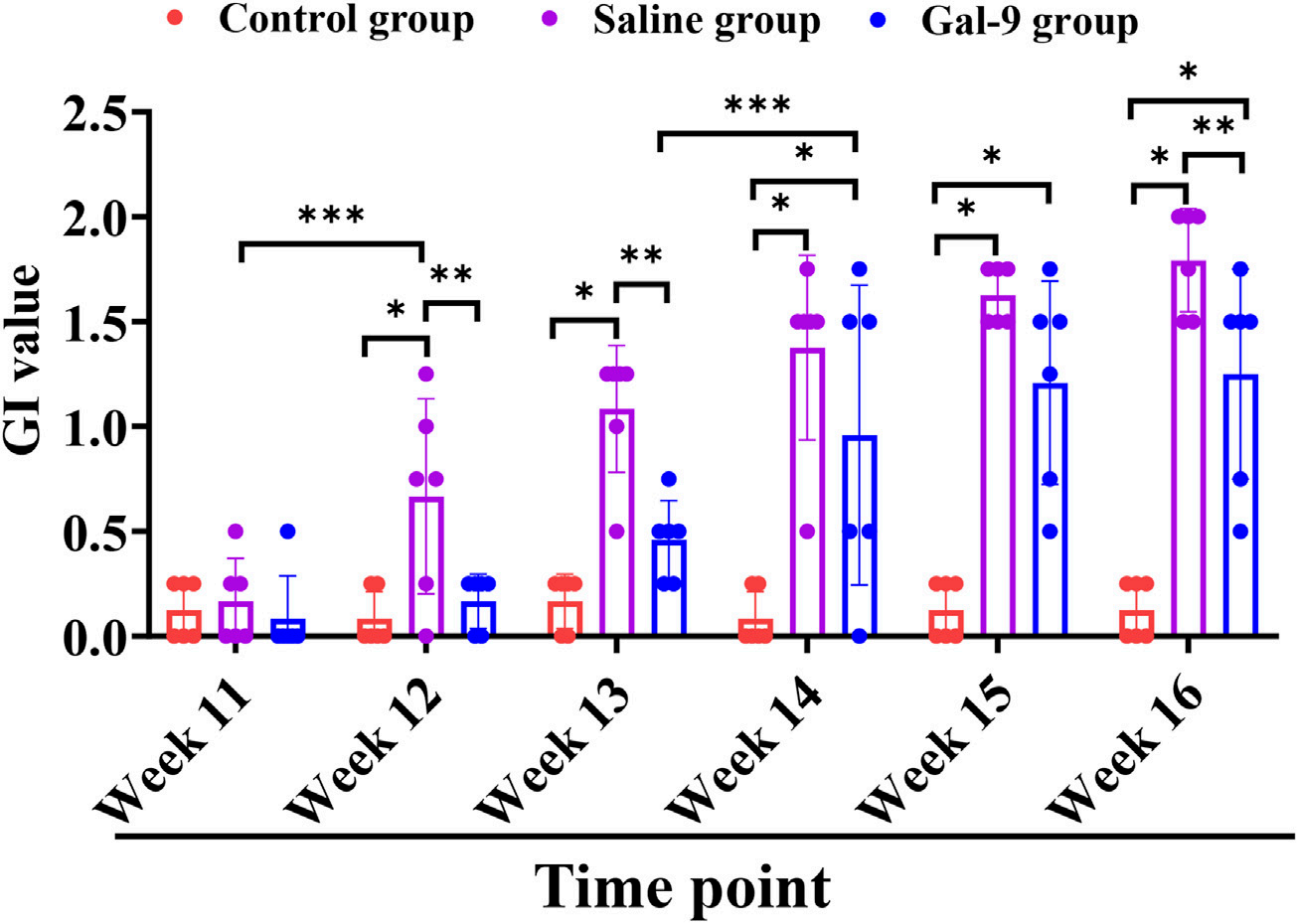
Comparison of GI among the Control, Saline, and Gal-9 groups. *P < 0.05, **P < 0.01, ***P < 0.001.

To evaluate the effects of Gal-9 on the inflammation induced by PI, we first observed the gross morphology of the implants and then monitored the GI value and BOP weekly. Substantial accumulation of plaque and food residues around the implants was evident in both the Saline and Gal-9 groups following PI induction (Figure 3). Analysis of the GI values (Figure 4) revealed that during weeks 12–13, the GI value in the Saline group was significantly higher those in the Control group and the Gal-9 group (*P* < 0.05); at weeks 14–15, both the Saline and Gal-9 groups exhibited significantly higher GI values compared to the Control group (*P* < 0.05); by week 16, the GI values in both the Saline and Gal-9 groups remained elevated in comparison to the Control group, with the Gal-9 group showing a lower GI value than the Saline group (*P* < 0.05). Notably, a significant increase in GI value was observed in the Saline group during weeks 11–12, while the Gal-9 group exhibited a significant rise in GI value during weeks 13–14 (*P* < 0.05). For the BOP positivity rate (Table 2), at 12 weeks and 16 weeks, the Saline group showed a higher positivity rate compared to the other two groups (*P* = 0.050).

**TABLE 2:** Comparison of BOP positivity rate.

Weeks	Control group		Saline group		Gal-9 group		χ^2^	*P*-value
	(+)	(−)	(+)	(−)	(+)	(−)		
11	0/6	6/6	3/6	3/6	1/6	5/6	4.818	0.09
12	0/6	6/6	4/6	2/6	2/6	4/6	6.000	0.050
13	1/6	5/6	5/6	1/6	4/6	2/6	5.850	0.054
14	1/6	5/6	5/6	1/6	4/6	2/6	5.850	0.054
15	1/6	5/6	5/6	1/6	5/6	1/6	7.481	0.024
16	2/6	4/6	6/6	0/6	4/6	2/6	6.000	0.050

Note: BOP, bleeding on probing; Gal-9, Galactin-9.

### 3.3 X-ray and micro-CT evaluation of alveolar bone absorption

**FIGURE 5:**
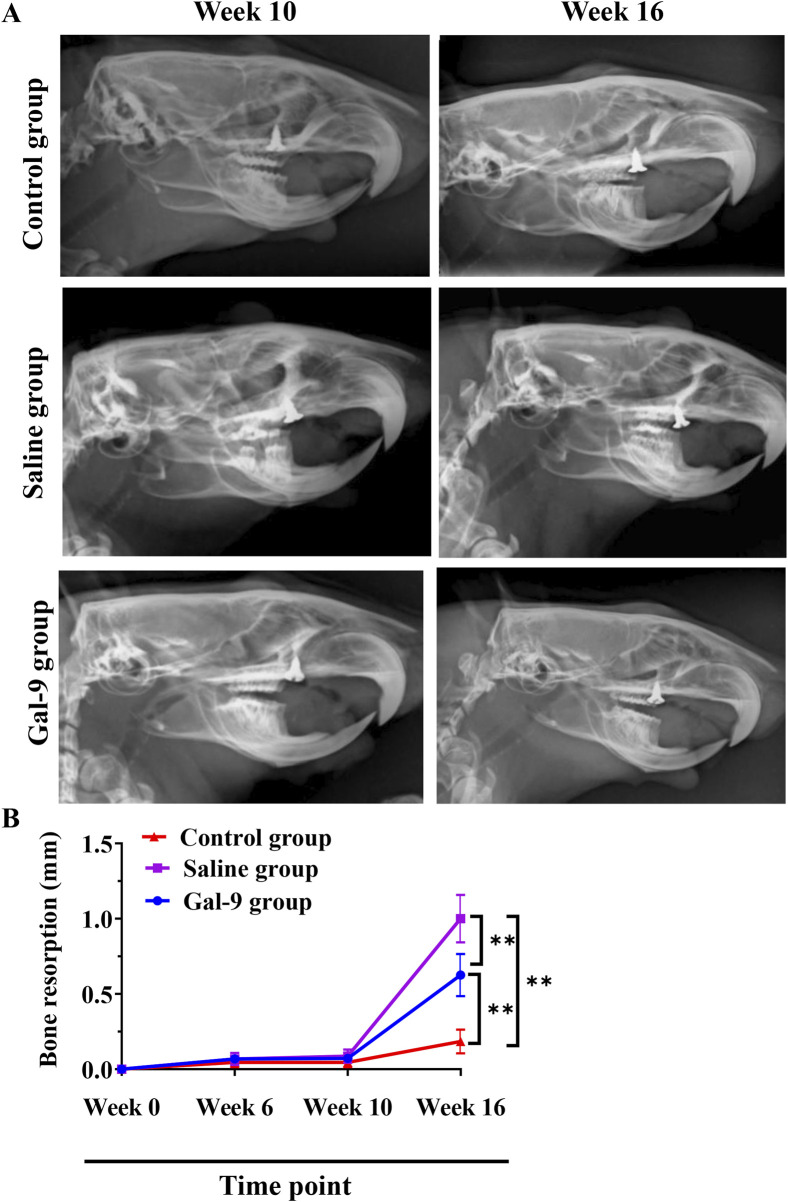
X-ray analysis of bone resorption. **(A)** Representative X-ray images at week 10 (before PI) and week 16 (after PI) in the Saline and Gal-9 groups. **(B)** Bone resorption at weeks 0, 6, 10, and 16. **P < 0.01.

To evaluate alveolar bone absorption, an X-ray examination was conducted at weeks 0, 6, 10, and 16. The representative X-ray images at week 10 and week 16 are presented in Figure 5A. Statistically, there was no notable alveolar bone absorption at weeks 0, 6, and 10 (Figure 5B). At week 16, the Saline group exhibited a significantly higher alveolar bone absorption rate compared to both the Gal-9 and Control groups (*P* < 0.05). The alveolar bone absorption rate in the Gal-9 group was also significantly higher than that in the Control group (*P* < 0.05).

**FIGURE 6:**
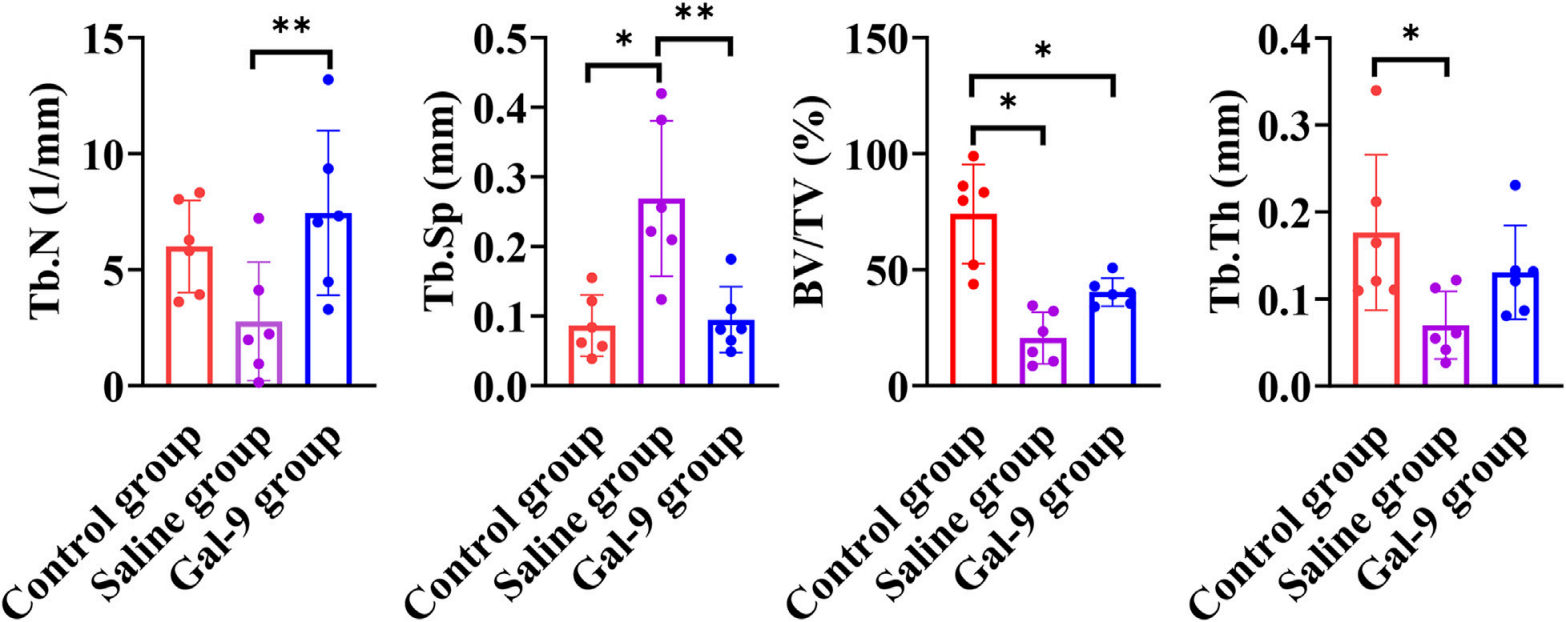
Comparison of TB.N, TB. Sp, BV/TV, and Tb/Th among the Control, Saline, and Gal-9 groups. *P < 0.05, **P < 0.01.

**FIGURE 7:**
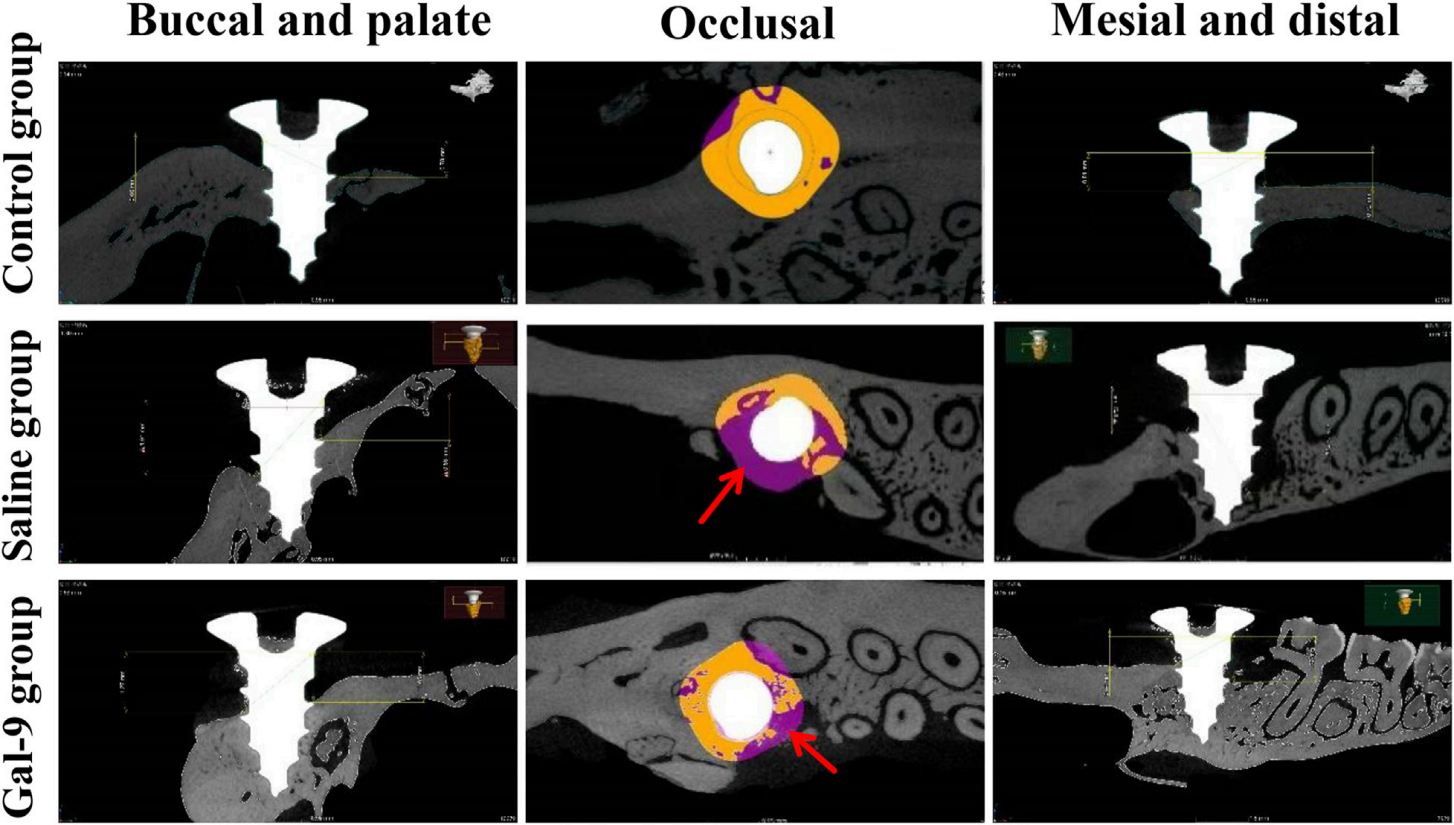
Degree of bone absorption and the area of bone integration around the implant. The micro-CT images are shown. The red arrows indicate the sites of bone absorption.

Furthermore, micro-CT analysis, as illustrated in Figure 6, revealed that the Tb.N of the Gal-9 group was significantly higher than that of the Saline group (*P* < 0.05). The Tb.Sp of both the Gal-9 and Control groups significantly decreased than that of the Saline group (*P* < 0.05). Moreover, the Gal-9 and Saline groups had significantly lower BV/TV than the Control group (*P* < 0.05). Additionally, the Tb.Th of the Saline group significantly decreased than that of the Control group (*P* < 0.05). However, there was no significant difference in the BV/TV and Tb.Th between the Saline and Gal-9 groups. Notably, on micro-CT images, well-integrated bone structures surrounding implants were observed in the Saline group, with minimal absorption evident in the buccal, proximal, and distal alveolar bone regions, and a broad area of bone integration (depicted in orange in Figure 7). In contrast, the Gal-9 group exhibited focal bone absorption around the implants, while the Saline group showed extensive absorption in the alveolar bone surrounding the implants (depicted in purple in Figure 7).

### 3.4 Proportions of M1 and M2 in peripheral blood

**FIGURE 8:**
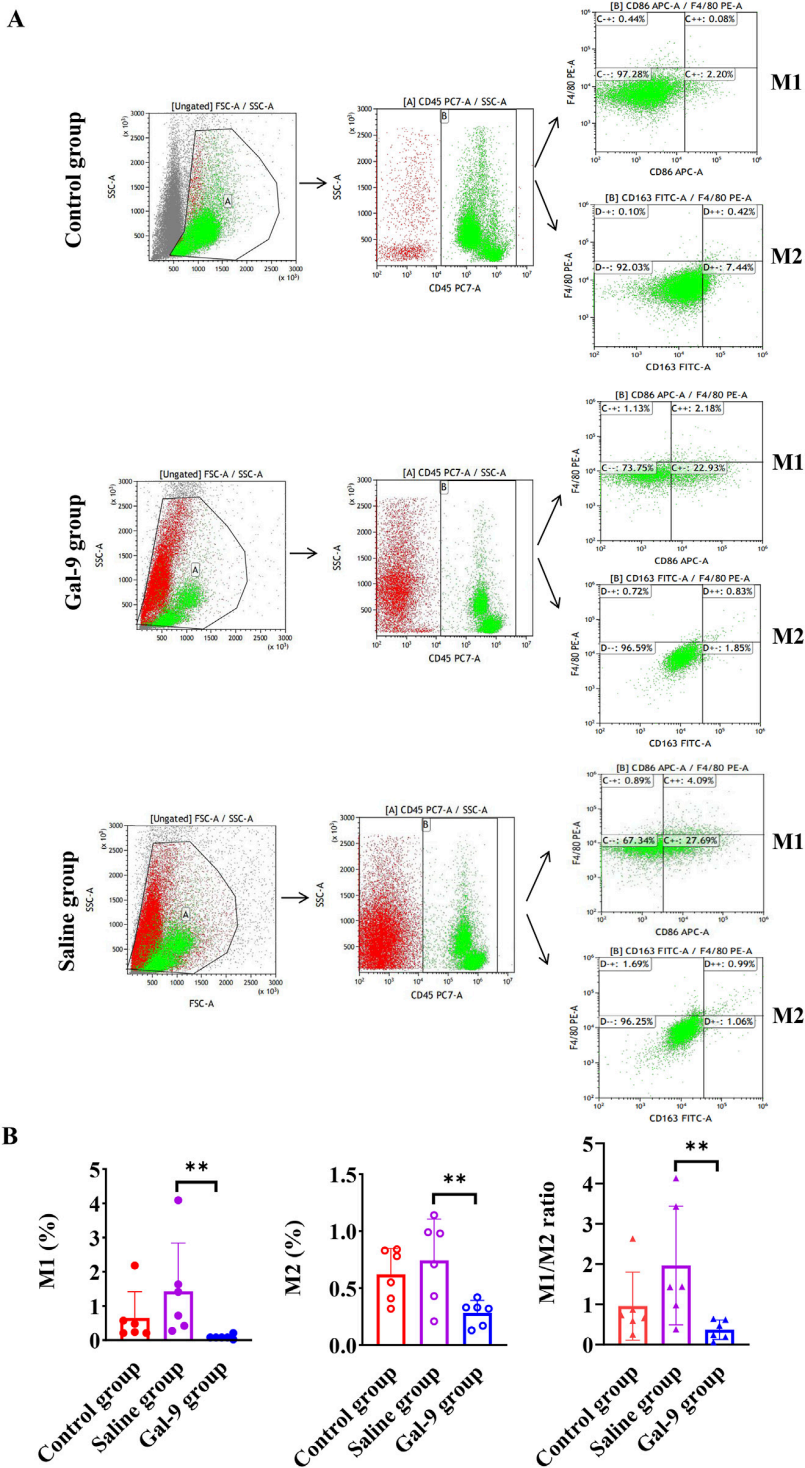
Flow cytometry analysis of macrophage proportion. **(A)** The gating strategies for M1 and M2 cells in each group. **(B)** The proportions of M1 and M2 and the ratio of M1 to M2. **P < 0.01.

Three groups of rats were subjected to flow cytometry analysis to determine the proportions of M1 and M2, as well as M1/M2 ratios in peripheral blood. The gating strategies for M1 and M2 in each group are presented in Figure 8A. It was found that the M1 and M2 proportions and the M1/M1 ratio in the Gal-9 group were significantly lower than those in the Saline group (*P* < 0.05) (Figure 8B).

### 3.5 Analysis of the correlation between micro-CT parameters, GI, and M1/M2 ratio

**FIGURE 9:**
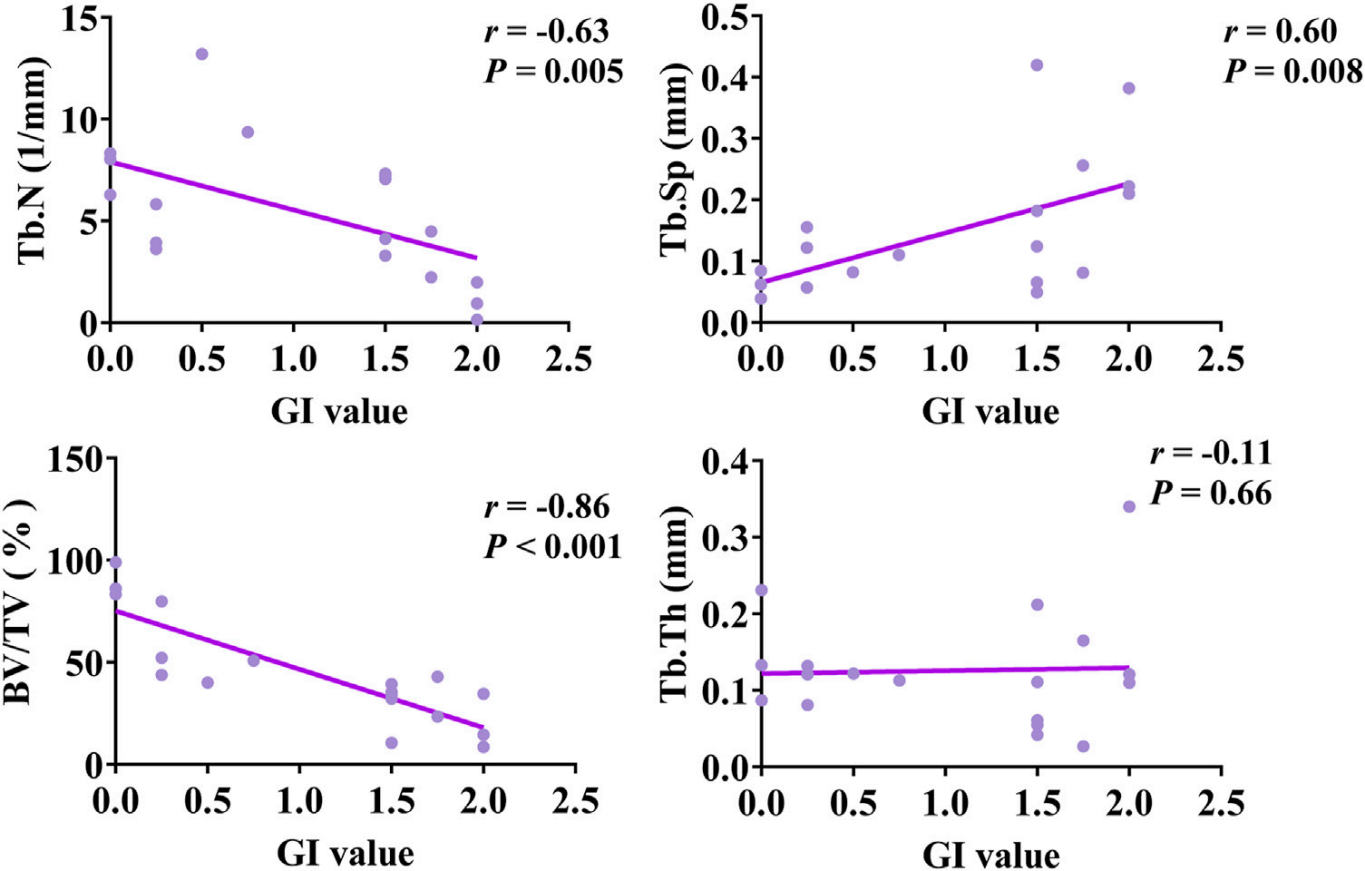
Spearman rank correlation analysis of GI value and micro-CT parameters.

**FIGURE 10:**
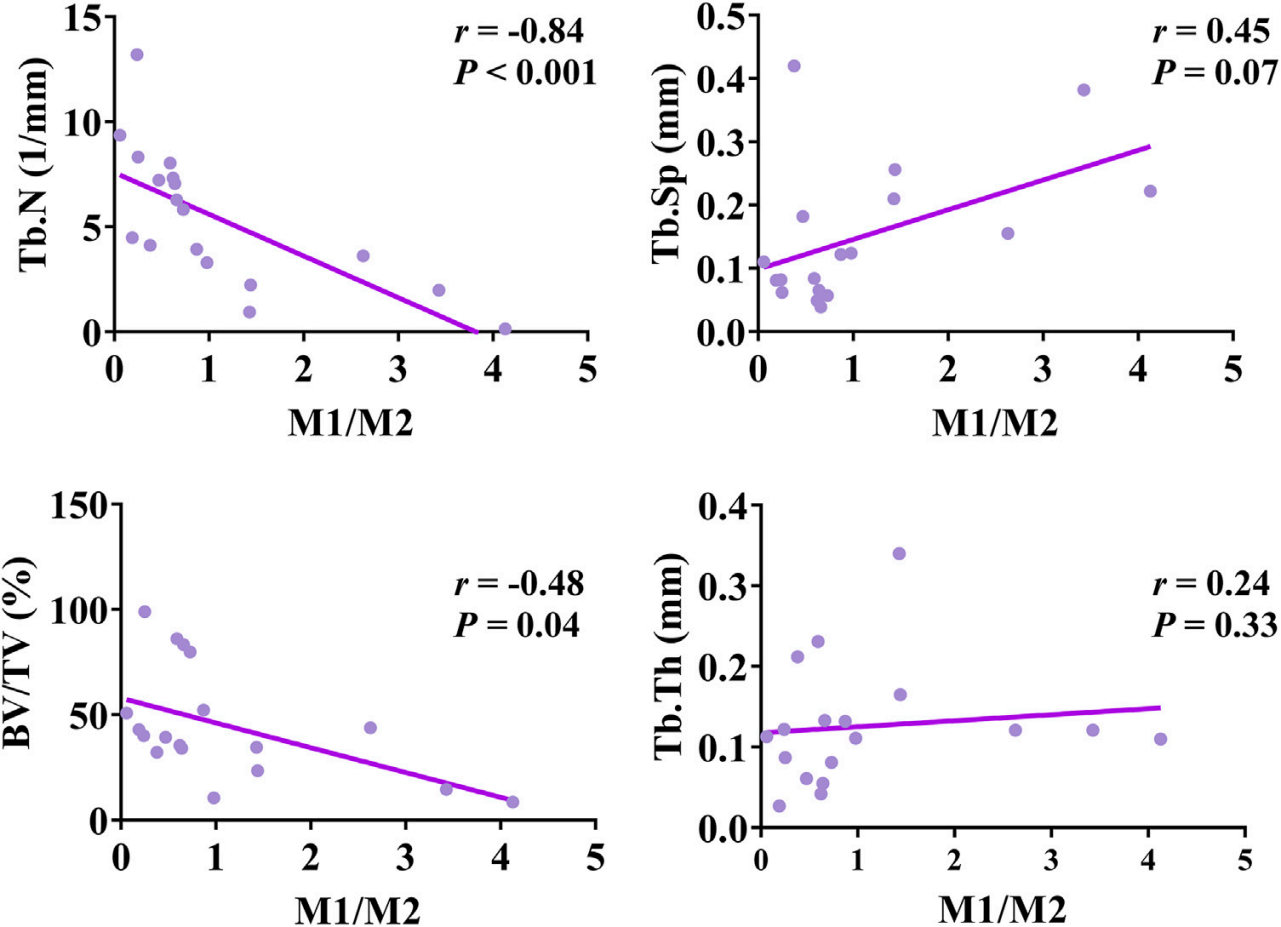
Spearman rank correlation analysis of M1/M2 ratio and micro-CT parameters.

The bivariate Spearman correlation showed that GI was negatively correlated with Tb.N (*r* = −0.63, P = 0.005), BV/TV (*r* = −0.86, *P* < 0.001), and Tb.Th (*r* = −0.11, *P* = 0.66), and positively correlated with Tb.Sp (*r* = 0.60, *P* = 0.008) (Figure 9). On the other hand, the M1/M2 ratio was negatively correlated with Tb.N (*r* = 0.84, *P* < 0.001) and BV/TV (*r* = −0.48, *P* = 0.04), but positively correlated with Tb.Sp (*r* = 0.45, *P* = 0.07) and Tb.Th (*r* = 0.24, *P* = 0.33) (Figure 10).

Multivariate linear regression analysis was conducted with GI and M1/M2 ratio as dependent variables and BV/TV, Tb.Th, Tb.N, and Tb.Sp as independent variables. The results showed that after adjustment for BV/TV, Tb.N, and Tb.Sp, Tb.Th still had a statistically significant impact on GI (*P* = 0.003) (Table 3). For each unit increase in Tb.Th, GI decreased by 0.021. In addition, Tb.Sp had a statistically significant impact on the M1/M2 ratio (*P* = 0.031) (Table 4). For each unit increase in Tb.Sp, M1/M2 ratio decreased significantly by 0.207.

**TABLE 3:** Multivariate linear regression analysis of factors affecting GI.

	Unstandardized coefficients		Standardized coefficients	t	*P*-value	Collinearity statistics	
	β	SE	β			Tolerance	VIF
Constant	2.185	0.455	—	4.247	0.001	—	—
BV/TV	0.587	1.435	0.084	0.409	0.689	0.475	2.106
Tb.Th	−0.021	0.006	−0.712	−3.728	0.003	0.547	1.829
Tb.N	−0.449	1.726	−0.043	−0.26	0.799	0.719	1.39
Tb.Sp	−0.039	0.043	−0.166	−0.916	0.376	0.606	1.651
R^2^	7.41						
Adjusted R^2^	0.66						
F	F = 9.268, *P <* 0.001						
Durbin-Watson	1.819						

Note: GI, gingival index; SE, standard error; VIF, variance inflation factor; TB.N, trabecular number; Tb.Sp, trabecular separation; BV/TV, bone volume/total volume; Tb.Th, trabecular thickness.

**TABLE 4:** Multivariate linear regression analysis of factors affecting M1/M2 ratio.

	Unstandardized coefficients		Standardized coefficients	t	*P*	Collinearity statistics	
	β	SE	β			Tolerance	VIF
Constant	2.552	0.905	—	2.819	0.014	—	—
BV/TV	1.18	2.853	0.114	0.414	0.686	0.475	2.106
Tb.Th	−0.007	0.011	−0.152	−0.59	0.566	0.547	1.829
Tb.N	−1.727	3.431	−0.113	−0.503	0.623	0.719	1.39
Tb.Sp	−0.207	0.086	−0.59	−2.415	0.031	0.606	1.651
R^2^	0.531						
Adjusted R^2^	0.386						
F	F = 3.672, *P* = 0.033						
Durbin-Watson	1.818						

Note: SE, standard error; VIF, variance inflation factor; TB.N, trabecular number; Tb.Sp, trabecular separation; BV/TV, bone volume/total volume; Tb.Th, trabecular thickness.

## 4 Discussion

Gal-9 protein, a promising immunotherapeutic agent, is used to modulate immune responses and manage immune-related disorders (Qi et al., 2019; Zhang et al., 2019a; Rodrigues Mantuano et al., 2020; Marino et al., 2023). It has been indicated that Gal-9 can influence endothelial cell growth, migration, and luminal formation, consequently promoting angiogenesis (Tang et al., 2019). Moreover, it also has a positive effect on alveolar bone regeneration and regulates the activation of immune cells and secretion of inflammatory factors, providing immune support for tissue repair and regeneration (Tang et al., 2019). In recent years, the function of Gal-9 in systemic inflammatory responses has been widely studied, showing effective preventive and therapeutic effects in inflammatory disease models (Du et al., 2023; Xiong et al., 2023). Xiong et al. demonstrated that exogenous Gal-9 alleviated colitis in mice (Xiong et al., 2023). The therapeutic effects of Gal-9 on lupus and rheumatoid arthritis have also been reported (Seki et al., 2008; Arikawa et al., 2009; Moritoki et al., 2013). Herein, this study analyzed the effects of prophylactic exogenous supplementation of Gal-9 protein on PI in rats from clinical, imaging, and immunological perspectives, providing new immune insights for its prevention and treatment.

First, this study recorded the changes in GI and found that the GI in the Saline group increased earlier than that in the Gal-9 group. At week 6, BOP, which serves as an indicator of periodontal tissue stability and may reflect the inflammatory status and activity levels of periodontal tissues in rats (Mombelli et al., 1995), was observed in the Saline group. Further analysis showed that the BOP positive rate in the Gal-9 group was lower than that in the Saline group. This result indicates that the clinical inflammation status and activity of rat periodontal tissues in the Gal-9 group are more stable than those in the Saline group. This is consistent with the results reported by Moritoki et al. (2013), who have confirmed that Gal-9 can alleviate the clinical severity of lupus. Therefore, this study preliminarily suggests that the Gal-9 protein may delay the onset of PI and alleviate the severity of clinical inflammation, thus positively impacting PI.

On the other hand, this study experienced a long experimental period and high operation difficulty, resulting in a significant loss of samples. Although there was PI induction in both the Saline group and the Gal-9 group, only the Saline group showed an increased implant loss rate after PI induction. This may be due to the prophylactic external supplementation of Gal-9 protein, which to some extent reduces the severity of inflammation, thereby reducing implant loss caused by inflammation. Interestingly, this study found that the implants in the Saline group of rats began to loosen and detach as early as the second week after PI induction, while the Gal-9 group started experiencing loosening and detachment from the fourth week. Therefore, we believe that the optimal period for inducing PI in rats should be within 4 weeks, which aligns with the viewpoint of Meng et al. (2023). Furthermore, changes in body weight can reflect the nutrition, mental state, and pain levels in rats. Our results showed that after inducing PI, there was a decrease in body weight in the Saline group, while the body weight of the Control group and the Gal-9 group continued to increase. We speculate that the Gal-9 group may have milder clinical symptoms and oral pain sensation compared to the Saline group, leading to better feeding and nutritional conditions in the Gal-9 group.

Imaging indicators are the gold standard for evaluating PI. In our study, the X-ray results revealed that bone resorption in the Gal-9 group was lower than in the Saline group. This result was consistent with GI value and BOP results, further confirming the beneficial role of Gal-9 protein in PI. To provide a more precise evaluation of the effect of Gal-9 protein on inflammatory responses surrounding implants and the consequential loss of supportive bone, micro-CT imaging was conducted. This analysis yielded the main indicators for assessing the morphological structure of trabecular bone space, including TB. N, Tb. Sp, BV/TV, and Tb.Th. According to bone morphometrics (Pei et al., 2008; Wang, 2010; Chen et al., 2014; Wei and Zhang, 2018), it can be demonstrated that bone resorption around implants in both the Saline group and the Gal-9 group rats was greater than in the Control group, while the synthetic metabolism of alveolar bone around implants in the Gal-9 group was greater than in the Saline group. It has been reported that the Gal-9 protein can influence the regenerative ability of alveolar bone tissue by regulating inflammatory reactions, stem cell proliferation and differentiation, and bone cell activity, thereby promoting alveolar bone reconstruction (Moriyama et al., 2014). Given the pivotal role of alveolar bone in implant support and repair, this study provides initial evidence that prophylactic exogenous supplementation of Gal-9 can enhance alveolar bone synthesis around implants, consistent with the findings of Moriyama et al. (2014).

Moreover, the destruction of the mucosa and alveolar bone around the implant interact and restrict each other. When there is inflammatory destruction of the alveolar bone around the implant, the GI will also change accordingly. In the present study, multivariate linear regression analysis on TB.N, Tb.Sp, BV/TV, and Tb.Th revealed that Tb.Th was the most important factor affecting the GI value. This suggests that future studies should focus on enhancing Tb.Th to mitigate clinical inflammation.

Notably, while the gold standard for assessing peri-implantitis in our study was through radiographic indicators (X-ray and micro-CT assessments), we included BOP and GI as supplementary indicators. However, due to the anatomical differences in the gingival sulcus between rats and humans, the direct application of these methods to rat models is subject to ongoing debate. The gingival structure in rats is simpler, and the connective tissue is thinner, which can lead to a higher propensity for bleeding during probing. These anatomical limitations may complicate the interpretation of clinical indices and could raise concerns regarding their reliability as indicators of periodontal health. Thus, the clinical relevance of our periodontal examination findings requires careful consideration. Nonetheless, they provide valuable insights that could benefit future investigations aimed at understanding the dynamics of periodontal inflammation in experimental settings.

It is well known that macrophages and their cytokines are effective in regulating the activities of osteoclasts and osteoblasts, and they are the main regulators of angiogenesis (Han et al., 2022; Yuan et al., 2023). Inflammatory factors released by M1 may worsen inflammation in the alveolar bone and affect tissue repair and regeneration, ultimately leading to the destruction of the alveolar bone and increased bone resorption. In contrast, the anti-inflammatory factors released by M2 help reduce inflammation, promoting the stability of the alveolar bone. Therefore, the transition from M1 to M2 is considered an important event in bone healing (Zhang et al., 2019b). In this study, the M1 and M2 proportions and the M1/M2 ratio in the Gal-9 group were lower than those in the Saline group, indicating a weaker inflammatory response in the Gal-9 group. Furthermore, the M2 proportion was higher than the M1 proportion in the Gal-9 group. This explains why in the Gal-9 group of rats, micro-CT showed better bone integration compared to the Saline group, with the generation of new bone trabeculae. Therefore, this study preliminarily confirms that prophylactic administration of Gal-9 protein can promote macrophage polarization from M1 to M2, thus alleviating inflammation, promoting tissue repair, and supporting the development and growth of new tissues. This is consistent with the results of Zhang et al., which demonstrated that Gal-9 regulated the polarization of macrophages derived from mice’s femoral bone marrow towards M2, playing an important role in regulating angiogenesis (Zhang et al., 2019b).

Additionally, this study conducted a correlation analysis between M1/M2 ratios and imaging parameters. The results revealed a negative relationship between Tb.N and BV/TV with M1/M2 ratios. Furthermore, Tb.Sp was identified as the primary factor influencing M1/M2 ratios, showing a consistent decrease in M1/M2 ratios with a decrease in Tb.Sp. These findings imply that enhancing Tb.Sp could potentially ameliorate PI symptoms. However, the underlying immunological mechanisms warrant additional elucidation.

In summary, the present findings suggest that the exogenous prophylactic supplementation of Gal-9 protein can modulate the polarization of macrophages, enhance their polarization towards M2, and ameliorate the M1/M2 ratio. Additionally, this supplementation facilitates angiogenesis and tissue reconstruction, resulting in the synthesis of alveolar bone surrounding the implant and the generation of new bone trabeculae. Consequently, it enhances the GI value of PI in rats, alleviates the extent of mucosal inflammation around the implant, and contributes to a certain degree to the prevention of PI disease. These results suggest that Gal-9 holds therapeutic potential in the prevention and treatment of PI. Further research into the immunomodulatory mechanisms of Gal-9 and its clinical applications in PI management are essential for advancing therapeutic strategies in oral implantology. While the presence and bioavailability of Gal-9 4 weeks post-administration have raised concerns, we plan to explore variations in the timing and frequency of administration to optimize its prophylactic effects against PI, as well as to refine our understanding of the mechanisms involved.

## Data Availability

The raw data supporting the conclusions of this article will be made available by the authors, without undue reservation.
